# Development of Multifunctional Hybrid Coatings (Mechanically Resistant and Hydrophobic) Using Methyltrimethoxysilane–Diethoxydimethylsilane–Tetraethoxysilane Mixed Systems

**DOI:** 10.3390/ma17020368

**Published:** 2024-01-11

**Authors:** Charlène Pellegrini, Sandrine Duluard, Marie Gressier, Viviane Turq, Florence Ansart, Marie-Joëlle Menu

**Affiliations:** Centre Interuniversitaire de Recherche et d’Ingénierie des Matériaux (CIRIMAT), Université Toulouse 3 Paul Sabatier, Toulouse INP, CNRS, Université de Toulouse, 118 Route de Narbonne, CEDEX 9, 31062 Toulouse, Francesandrine.duluard@univ-tlse3.fr (S.D.); marie.gressier@univ-tlse3.fr (M.G.); viviane.turq@univ-tlse3.fr (V.T.); florence.ansart@univ-tlse3.fr (F.A.)

**Keywords:** sol-gel coating, hydrophobic, silicon oil, mechanical properties, mechanical resistance

## Abstract

For many industrial applications, the simultaneous presence in a material of different functional properties is necessary. The main interest lies in making a single material more versatile and durable, less fragile and more efficient. In this study, two concomitant properties in the same material were mainly studied: resistance to cracking and the increase in its hydrophobic properties. The chosen process was the sol-gel route due to its versatility and the ease of formulating materials from various precursors in order to obtain (multi)functional materials. In this paper, sol-gel coatings were prepared with tetraethoxysilane, methyltrimethoxysilane and diethoxydimethylsilane as precursors. Tetraethoxysilane was mainly used to improve the material’s mechanical properties, especially hardness, and silicon oil was added to improve its hydrophobic behavior. The integration of silicon oil was monitored via ^29^Si NMR. Microstructural characterizations were carried out to correlate the multi-scale properties with the microstructure of the derived films. Young’s modulus and hardness were measured to highlight the effect of key formulation parameters on the mechanical strength of the coatings. The synergistic effect of these precursors is underlined as well as the beneficial effect of silicon oil (generated in situ or precondensed).

## 1. Introduction

Organic–inorganic hybrid sol-gel coatings based on siloxane–oxide systems are very promising for many applications, such as anti-corrosion [1], anti-friction or anti-wear [2,3,4,5] coatings, for example, but also for the development of hydrophobic coatings [6,7,8]. Hybrid networks are obtained by mixing inorganic precursors such as tetraethoxysilane (TEOS), zirconium tetrapropoxide (ZrTPO) or titanium tetrapropoxide (TiTPO) and various organosilanes, including the well-known glycidoxypropyltrimethoxysilane (GPTMS), 3-methacryloxypropyltriethoxysilane (MAPTES) and methyltrimethoxysilane (MTMS). Diethoxydimethylsilane (DEDMS) is also well known for either obtaining hydrophobic protective coatings [6] or for processing metallic prostheses for tissue engineering applications [9,10]. Hybrid materials have given rise to numerous fundamental characterizations, which make it possible to link the chemical structure and microstructure to the working properties in working conditions. In our study, the hybrid material skeleton occurs mainly due to the MTMS network, and various quantities of TEOS or DEDMS are being added in order to improve the mechanical and/or hydrophobic properties.

Thus, Babonneau et al. studied TEOS/DEDMS and TEOS/PDMS-OH gels, where PDMS-OH is represented by OH-terminated poly(dimethyl)siloxane chains [11]. The structural evolution of this system has been followed via NMR from the molecular precursors to the final materials. Co-condensation reactions between PDMS-OH or precondensed DEDMS and TEOS have been highlighted via ^29^Si NMR.

Deng et al. studied ORMOSIL TEOS/DEDMS compositions [12] and show that co-condensation between TEOS and DEDMS monomers occurs in acidic conditions. These result are in agreement with those of Babonneau. Richards et al. studied the antifouling potential of TEOS or DEDMS coatings [13]. They found that using TEOS as a precursor permits achieving interesting hydrophobic properties with a water contact angle > 100°. DEDMS-based coatings are also promising, with a water contact angle around 100°. Gavilan et al. studied the use of MTMS/TEOS, DEDMS/TEOS and PDMS-OH/TEOS sol-gel coatings on stainless steel for bone tissue engineering applications [9,10]. They concluded that the chemical structure obtained using the DEDMS precursor shows an interesting balance between protection and bioactivation and that PDMS/TEOS has the highest hydrophobic behavior.

Salazar-Hernandez et al. studied TEOS/PDMS-OH/colloidal silica sol-gel in stone consolidation [14]. It appeared that PDMS-OH improves elasticity thanks to siloxane chains chemically bonded to the inorganic silica skeleton (TEOS and colloidal silica). This phenomenon has been confirmed via ^29^Si NMR. Malzbender et al. studied the mechanical properties of a MTMS/TEOS sol-gel coating filled with alumina or silica particles [15]. They measured a Young’s modulus of 5 GPa and a hardness of 0.7 GPa for a coating without particle addition. Ballarre et al. [16] measured a Young’s modulus of 6.5 GPa and a hardness of 0.92 GPa for a hybrid silica-based coating with TEOS and methyltrimethoxysilane (MTES) as precursors.

In this paper, the main aim of our study is to develop new MTMS-based coatings combining both hydrophobic and scratch-resistance properties. The matrix of the coating is based on an MTMS-based siloxane network associated, or not, with TEOS, which is known in the literature to improve coatings’ mechanical properties [17]. DEDMS is introduced in order to improve hydrophobic properties through the following two different and original routes: either as a silicone oil after precondensation via hydrolysis of the DEDMS or directly as a silane precursor for in situ oil generation.

The integration of silicone oil is studied via NMR analysis coupled with SEM pictures of the surface. Its hydrophobic behavior is characterized by static and dynamic contact angle measurements. The mechanical properties are evaluated via nanoindentation.

These multi-scale characterizations are then correlated with the various formulations and finally compared in order to understand the effects of process parameters.

## 2. Materials and Methods

### 2.1. Materials

The first step involved preparing a sol from the following precursors. Methyltrimethoxysilane (MTMS, 98%), tetraethoxysilane (TEOS, 98%), diethoxydimethylsilane (DEDMS, 97%), glacial acetic acid (CH_3_CO_2_H, 99%) and 2-propanol (iPrOH, 99.5%) were purchased from Sigma-Aldrich (Saint-Quentin Fallavier, France), and 2-butoxyethanol (C_4_H_9_OC_2_H_4_OH, 99%) was obtained from Honeywell. All reagents were used without further purification. The roles and semi-developed formulas of the silanes used as precursors are indicated in Table 1.

All coatings were applied on sandblasted and dried aluminum substrates. Spray deposition was then performed with an air pressure of 4 bars with manual handled pulverization device using a high-quality SRI Pro Lite spray gun (Larius France, Estrablin, France). In order to compare all the samples in similar conditions, areal mass densities were controlled after deposition. An average value of 1.4 mg/cm^2^ (+/−0.2) was measured; this deposited mass is sufficient to ensure a thickness higher than 5 microns. After deposition, a thermal treatment at 300 °C for 10 min was applied. To complete the study, xerogels were obtained from sols with the same heat treatment as for coatings.

### 2.2. Polycondensation of Diethoxydimethylsilane, pDEDMS

The precondensation parameters of DEDMS to obtain a silicone oil were selected according to the publication of Kalinina et al. [18] who studied the condensation of DEDMS in active medium using several conditions (water and acetic acid contents, temperature, reaction time). A mixture of 1.3 g (0.070 mol) of water, 4.0 g (0.070 mol) of acetic acid and 2.0 g (0.013 mol) of DEDMS was stirred 4 h at room temperature. According to the literature, in these conditions, the reaction yielded 50 wt.% of linear polymer HO(CH_3_)_2_SiO[Si(CH_3_)_2_O]nSi(CH_3_)_2_OH of 1600 Da and 50 wt.% of cyclic oligomers where [(CH_3_)_2_SiO)]_4_ D4 and [(CH_3_)_2_SiO)]_5_ D5 oligomers predominate in 37% and 11% proportions, respectively. The resulting silicone oil will be referred to as pDEDMS for precondensed DEDMS. The ^29^Si{^1^H} NMR spectrum of the silicone oil is presented in Figure S1 in the Supplementary Material. In this spectrum, three types of silicon atoms appear distinctly; they can be assigned according to published data on organosilicon linear polymers [19,20,21]: D^1^ units in (CH_3_)_2_(HO)Si*-O-Si(CH_3_)_2_- exhibit chemical shifts ranging from −10.5 (n = 0) to −12.5 (n > 5), while D^2^ units in -O-(CH_3_)_2_Si*O)_n_ appear in the range from −20 to −22 ppm. The presence of small rings is confirmed by the signal at −18.1 ppm, which is characteristic of silicon atoms in D4 cyclic oligomers as also described by Kalinina et al. [18], Babonneau [22] and Suda [20]. The signal at −7 ppm, fine and of low intensity, does not correspond to any of the small cyclic oligomers (D3 at −9 ppm); thus, it is not assigned.

### 2.3. Preparation of Sols and Xerogels

Six formulations are studied in the following, and molar proportions are detailed in Table 2. The first one is the “reference” formulation, M100, which is based on hydrolysis and the condensation of MTMS in acidic medium (acetic acid) without any additive. In the other formulations, DEDMS is introduced for the ex situ or in situ generation of dimethylsiloxane oligomers as oil; in the last case, co-condensation of DEDMS with MTMS should be considered. This is investigated via the introduction of a small amount of precondensed DEDMS either in the reference formulation (M100-pDEDMS) or in a reference formulation with the substitution of 25% of MTMS by DEDMS (M75-D25-pDEDMS). The influence of TEOS in these formulations was evaluated by adding a large amount of TEOS in the formulations (nearly 1/1 vs. MTMS) (T-M100, T-M100-pDEDMS and T-M75-D25-pDEDMS). In these last cases, the hydrolysis rate was increased to favor the hydrolysis of all the silane functions. All formulations are acidified with acetic acid either added during the precondensation of the DEDMS or directly for the M100 and T-M100 formulations.

In all formulations, 2-propanol and 2-butoxyethanol are systematically introduced to obtain 10 wt.% for each in the formulations. Sols are prepared according to the protocol detailed below for the M100 and T-M75-D25-pDEDMS formulations; the preparation is similar for the other compositions.

For the M100 formulation, 2.7 g (0.045 mol) of acetic acid was added to 19 mL (1.05 mol) of water and stirred 2 min; then, 57.6 mL (0.40 mol) of MTMS was introduced and stirred 2 min more. A mixture of 13.2 mL of 2-propanol (0.17 mol) and 11.2 mL (0.087 mol) of 2-butoxyethanol was added, and the sol was stirred 2 h at room temperature. The resulting sol was aged during 24 h. The composition MTMS:H_2_O:CH_3_CO_2_H:2-propanol:2-butoxyethanol is 55:19:3:10:10 in wt.% and 1:2.6:0.1:0.42:0.21 in molar ratios.

For the T-M75-D25-pDEDMS formulation, 2 g of p-DEDMS, 27.7 g (0.131 mol) of TEOS, and 26.4 g (1.47 mol) of water were stirred for 2 min. MTMS (15.8 g, 0.114 mol) and DEDMS (5.3 g, 0.036 mol) previously mixed were then introduced, and the mixture was stirred 2 min more. Finally, 2-propanol (8.8 g, 0.145 mol) and 2-butoxyethanol (5.3 g, 0.073 mol) complete the sol, which is stirred 2 h at room temperature and then left to mature for 24 h. The composition MTMS:H_2_O:CH_3_CO_2_H:TEOS:DEDMS:pDEDMS:2-propanol:2-butoxyethanol is 55:19:3:10:10 in wt.% and 1:2.6:0.1:0.42:0.21 in molar ratios.

### 2.4. Characterization Techniques

The chemical structure of the coating constituted of siloxane network incorporating in situ synthesized silicon oil has been followed by ^29^Si solid state NMR. The measurements were made on xerogels which underwent the same heat treatment as the coatings. ^29^Si cross-polarization magic angle spinning nuclear magnetic resonance (^29^Si CP MAS NMR) spectra were recorded using a Bruker Avance III 400WB (9.4 T) spectrometer (Bruker Biospin, Fällanden, Switzerland). Chemical shift references are tetramethylsilane (TMS), and a 4 mm zirconia rotor was used. MAS and CP MAS experiments were recorded. The rotation speed around the magic angle (MAS) was 8 kHz, and experiments were realized at room temperature. The observed silicon units were designed according to the usual notation employed in silicon chemistry: Q [SiO_4_], T [SiCO_3_], D [SiC_2_O_2_], and M [SiC_3_O]. The number added as a superscript (Q^n^, T^n^ or D^n^) indicates the number of oxo bridging bonds to the corresponding Si site [23]. Data were processed using “MNova 11.0” software; deconvolution was completed using DmFit software (version # 20200306) [24].

**Figure 1:**
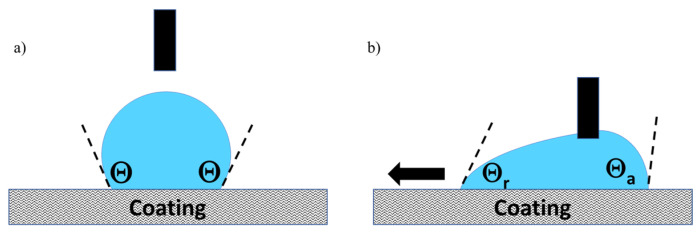
Static (**a**) and dynamic (**b**) contact angle method.

SEM observations were performed with a FEG JEOL JSM 6700 F SEM (JEOL, Croissy sur seine, France) with a 5 kV operating voltage and back-scattered electrons observation mode. The contact angle has been measured with a drop shape analyzer KRUSS at room temperature. Static contact angles and hysteresis was measured with a method described in the literature [25], which is presented in Figure 1a,b. The measurement is made on a flat sample, the syringe holds the water drop, and the plate moves, causing the movement of the drop. The deformation of the drop and therefore the drop adhesion force instrument is quantified by Δθ, the difference between the advancing θ_a_ and receding θ_r_ contact angles.

Roughness has been determined by confocal microscopy for all the samples using a “non-contact 3D optical profiler” S-neox (Sensofar) microscope (Sensofar metrology, Barcelona, Spain) used in confocal mode. The areal roughness parameter of arithmetic average of profile height deviation from the mean line, S_a_ parameter, lies in the range (0.5; 1.5 µm), indicating slightly rough coatings.”

Nanoindentation tests were performed with an UltraNanoIndenter (CSM Instruments, Anton Paar, Les Ulis, France) at a maximal normal load of 2 mN, corresponding to a penetration depth of about 500 nm in the coating. The loading and unloading rates were 4 mN.min^−1^. At least 3 indentation tests have been conducted for each sample. As the average thickness of all studied coatings lies above 5 µm, the ratio of the maximum penetration depth to coating thickness is smaller than 10%, thus ensuring that the substrate effect on the measured properties can be considered as negligible. A Berkovich indenter was used, and analysis was conducted using Oliver and Pharr’s method [26]. A Poisson ratio of 0.3 was used for the calculation of the mechanical properties.

## 3. Results and Discussion

In this part, the various results corresponding to the coatings without TEOS (with or without DEDMS) are first of all presented (surface micrographs and ^29^Si MAS and CP MAS NMR spectra).

After that, the results relative to the formulation including TEOS are then compared in terms of microstructural analyses and chemical analyses by ^29^Si NMR spectra (chemical shifts and proportion).

### 3.1. MTMS Reference Formulation Coatings (M100)

**Figure 2:**
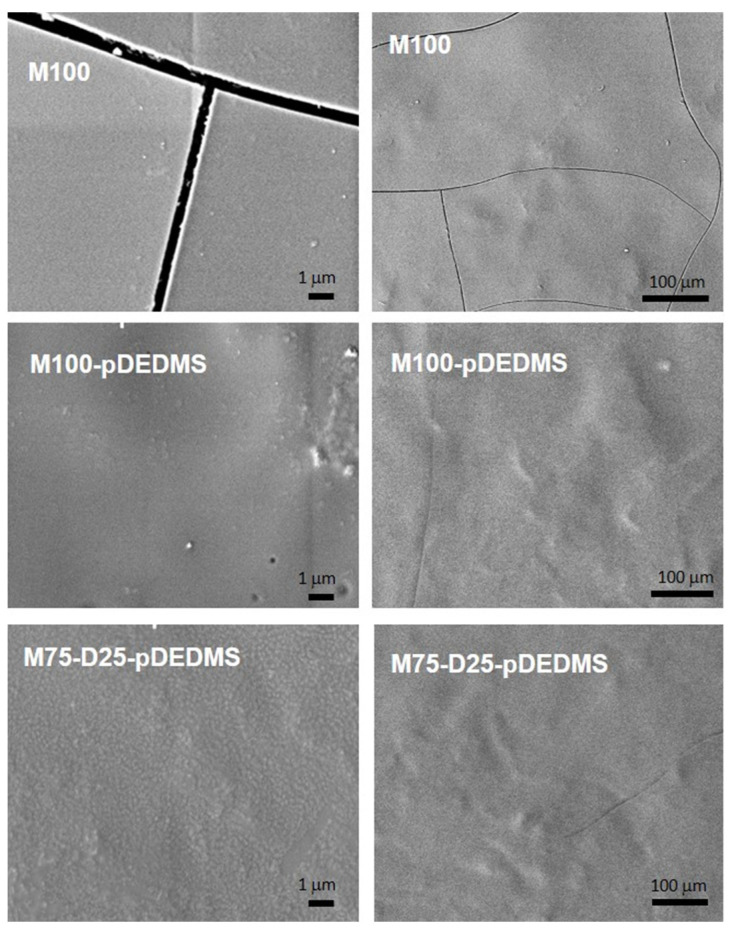
SEM images of coatings without TEOS: M100; M100-pDEDMS and M75-D25-pDEDMS (two magnifications).

The hydrolysis of MTMS in an acetic acid medium leads to a transparent sol after 24 h of aging. This sol is sprayed on 4000 series aluminum alloy substrates. The coatings obtained after drying are homogeneous and covering but reveal cracks, as observed in the micrographs in Figure 2 for M100. Cracks are probably formed during the rapid cooling after the curing step.

**Figure 3:**
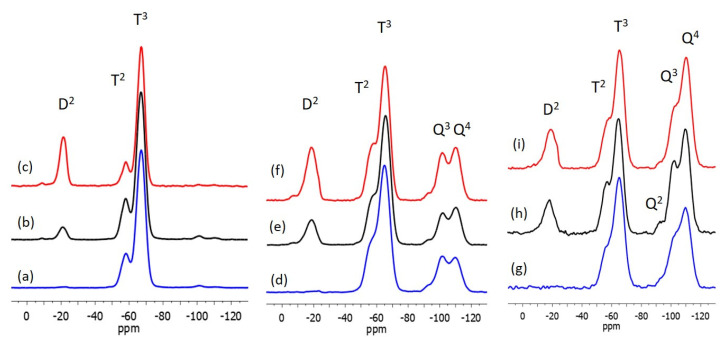
^29^Si CP MAS NMR of xerogels corresponding to (**a**) M100; (**b**) M100-pDEDMS; (**c**) M75-D25-pDEDMS; (**d**) T-M100; (**e**) T-M100-pDEDMS and (**f**) T-M75-D25-pDEDMS coatings and ^29^Si MAS NMR of xerogels corresponding to (**g**) T-M100; (**h**) T-M100-pDEDMS and (**i**) T-M75-D25-pDEDMS coatings.

In the ^29^Si NMR study, the assignment of peaks in the different chemical shift ranges was determined based on works described on systems involving MTMS, TEOS and/or DEDMS [19,20,22,23,27]. The inorganic network of the M100 reference coating is only made up of siloxane bonds provided by MTMS. The two resonances observed in the ^29^Si CPMAS NMR spectrum, Figure 3a, Table 3, at −58.2 and −67.1 ppm are assigned to the T^2^ and T^3^ silicon atoms, respectively, of the MTMS not completely condensed. The higher proportion of T^3^ type silicon atoms is evident in the spectra and is confirmed by the deconvolution of the peaks, which gives a proportion of 19% T^2^ silicon atom and 81% T^3^ silicon atom. It is interesting to see that after a heat treatment of the coating of only 10 min at 300 °C, the condensation of the siloxane network is almost complete (93.7%, see Table 4), which is surely inherent to the structure of the MTMS precursor consisting of only the T silicon atom type.

### 3.2. DEDMS Incorporation in MTMS Based Coatings

DEDMS is introduced in the MTMS formulation either as a molecular precursor and/or as a precondensed DEDMS (pDEDMS) silicone oil. The introduction of only 2 wt.% of pDEDMS in the formulation M100-pDEDMS is enough to improve the properties of the coating, since the surface of the coating appears without any cracks, as it can be seen in Figure 2. In the ^29^Si CPMAS NMR spectrum, as shown in Figure 3b, very few changes were observed since the T^2^ and T^3^ signals are always present at −57.9 and −67.0 ppm with almost the same proportion as in M100. The presence of pDEDMS slightly increases the T^2^:T^3^ proportion to 21:79 in favor of T^2^, indicating a slight reduction in the condensation of the siloxane network. The D^2^ signal due to the silicon atoms of the precondensed pDEDMS (SiC_2_O_2_) is visible around −21.0 ppm (two resonances at −19.4 and −21.8 ppm, Table 3) assigned, respectively, to D^2^ units of short-chain linear polymers (n > 5), HO(CH_3_)_2_SiO[Si*(CH_3_)_2_O]_n_Si(CH_3_)_2_OH [28]. Silicon atoms of the D^1^ unit are expected to appear between −11 and −13 ppm, so an absence of resonance in this range indicates that the pDEDMS is linked to the siloxane network of the MTMS.

A new experiment was evaluated by introducing a large proportion of DEDMS as a precursor directly mixed with MTMS with the same amount of pDEDMS as in the M100-pDEDMS. This formulation corresponds to the M75-D25-pDEDMS coating whose molar proportions are detailed in Table 2. The coating is also covering and homogeneous, without cracks, revealing a relatively homogeneous nanostructure. This behavior could be due to the pDEDMS as already observed in the previous experiment. It is also expected that free DEDMS of this experiment would be co-condensed with the main organosilane MTMS, since it is introduced at the same time during the preparation of the sol leading to a higher flexibility of the network, and it also condensed itself for the in situ generation of a short chain of polydimethysiloxane oil, leading also to the same effect as observed for pDEDMS.

**Figure 4:**
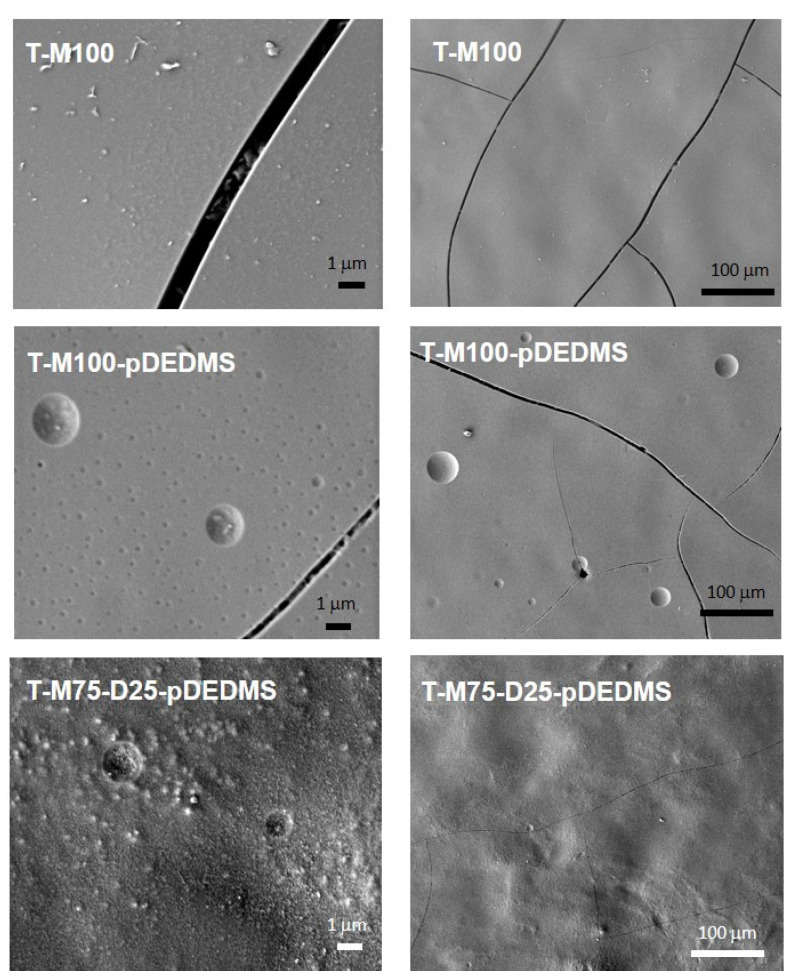
SEM images of coatings containing TEOS: T-M100, T-M100-pDEDMS and T-M75-D25-pDEDMS (2 magnifications).

The ^29^Si CP MAS NMR spectrum of the M75-D25-pDEDMS sample is presented in Figure 4c. Three types of silicon atoms clearly appear at −21.3, −58.4 and −67.3 ppm (see Table 3). In the low field range, the signal corresponds to the D^2^ silicon atom of the short chain of the p-DEDMS. The presence of DEDMS in the bulk of the matrix is highlighted with the modification of the proportions of the T^2^ and T^3^ signals, respectively, at −58.4 and −67.3 ppm. The presence of DEDMS led to a slight increase in the proportion of T^3^ and therefore to better condensation of the siloxane network; the condensation degree of MTMS increases from 93.5 to 95.7% (Table 4). Furthermore, this indicates that the DEDMS also condenses with the MTMS modifying the structure of the hybrid matrix, creating different T^3^ units (T^3^-D^2^-T^3^ or T^3^-D^2^-T^2^).

### 3.3. Effect of TEOS Addition on the MTMS and MTMS/DEDMS Formulations

Based on the previous MTMS/DEDMS coatings, the introduction of TEOS into M100; M100-pDEDMS and M75-D25-pDEDMS formulations was investigated to evaluate the possibility of improving the mechanical properties of the coatings. The composition of these formulations is detailed in Table 2. TEOS was introduced in close to 1:1 molar amounts for MTMS:TEOS in all formulations. SEM images of the surface of the three corresponding coatings are presented in Figure 4 at two magnifications. In both cases, the coatings appeared covering and crack-free.

Round shape elements with a diameter of several microns appear on the coating, which is probably due to a heterogeneous incorporation of the DEDMS silicon oil from pDEDMS or from in situ condensed DEDMS at a submicronic scale.

In T-M100-pDEDMS coatings containing TEOS, the substitution of 25%mol of the MTMS by the DEDMS precursor, i.e., T-M75-D25-pDEDMS, makes the surfaces more heterogeneous (Figure 4).

The ^29^Si CP MAS NMR spectra of the T-M100-pDEDMS and T-M75-D25-pDEDMS samples are presented in Figure 3e,f together with the corresponding MAS experiments in Figure 3h,i. Spectra of the T-M100 sample are also given for comparison (Figure 3d,g). The data, chemical shifts and proportions obtained by deconvolution are reported in Table 3. The degrees of condensation of the three different organosilanes are calculated using the proportions obtained by the deconvolution of the ^29^Si MAS NMR spectra, and the values are summarized in Table 4.

The introduction of TEOS on the M100 formulation and coating increases the rigidity of the network as expected with this precursor capable of potentially creating four siloxane bonds. Even if the networks remain well condensed (>85% for TEOS and >90% for MTMS), the degree of condensation of the MTMS clearly decreases in the presence of TEOS from 93.7% to 91.6%, showing that MTMS and TEOS react well together. The co-condensation of MTMS and TEOS in T-M100 is also demonstrated by the low field shift of the T2 and T3 signals from −58.2 and −67.1 ppm, for xerogels without TEOS, to −56.3 and −65.1 ppm in T-M100.

In the ^29^Si CP MAS NMR spectrum of T-M100-pDEDMS (Figure 4e), again, signals are observed in the three ranges of chemical shifts corresponding to the three types of silicon atoms of DEDMS, MTMS and TEOS precursors. These signals appear at –18.5 ppm (D^2^), −57.2 (T^2^), −65.6 (T^3^), −94.1 (Q^2^), −101.9 (Q^3^), and −110.3 ppm (Q^4^). These last two peaks are characteristic of silicon atoms of Q^3^, SiO_3_(OH), and Q^4^ units, SiO_4_, which are usually observed for an incompletely condensed siloxane network obtained by the hydrolysis of TEOS. All condensation degrees were calculated from the deconvolution of the ^29^Si MAS NMR experiments, which is especially important for the degree of condensation of the TEOS, which is minimized in the CP MAS experiments. In the absence of TEOS in the formulation, the MAS and CPMAS spectra are really very similar, which is why only the MAS spectra of T-M100, T-M100-pDEDMS and T-M75-D25-pDEDMS samples are presented, as shown in Figure 3g–i, respectively. The amount of Q^2^, SiO_2_(OH)_2_ units is always very low (1.1%), and the degree of condensation of TEOS greatly increases from 85% in T-M100 to 91.3% in T-M100-pDEDMS. These changes in the NMR spectrum highlight the effect of pDEDMS, which seems to react with the siloxane network constituted by TEOS. The reaction of DEDMS with TEOS leads to a more condensed network more easily (fewer reactive sites required) than the MTMS-TEOS reaction. This can be seen in the slight decrease in the condensation degree of MTMS (91.6 to 90.6%). The T^2^:T^3^ ratio further evolves in favor of T^2^ as already observed when DEDMS MTMS co-condensation occurs. In T-M100-pDEDMS, the T^2^:T^3^ ratio reaches 14.5:37.1.

When DEDMS is introduced in a larger amount, T-M75-D25-pDEDMS, the same effect is observed with condensation degrees of MTMS and TEOS (90.2 and 90.8%, respectively) higher than those of T-M100 for TEOS and lower for the condensation of the MTMS. In T-M75-D25-pDEDMS NMR spectra (Figure 3f,i), even if an additional resonance is observed in the D^2^ units range at −22.9 ppm (0.9%) indicating the presence of a few longer linear polymer chains, the presence of D^2^ units (Q-D^2^-Q or D-D^2^-Q) at −19 and −13 ppm highlights the co-condensation of DEDMS and TEOS in the siloxane network.

**Figure 5:**
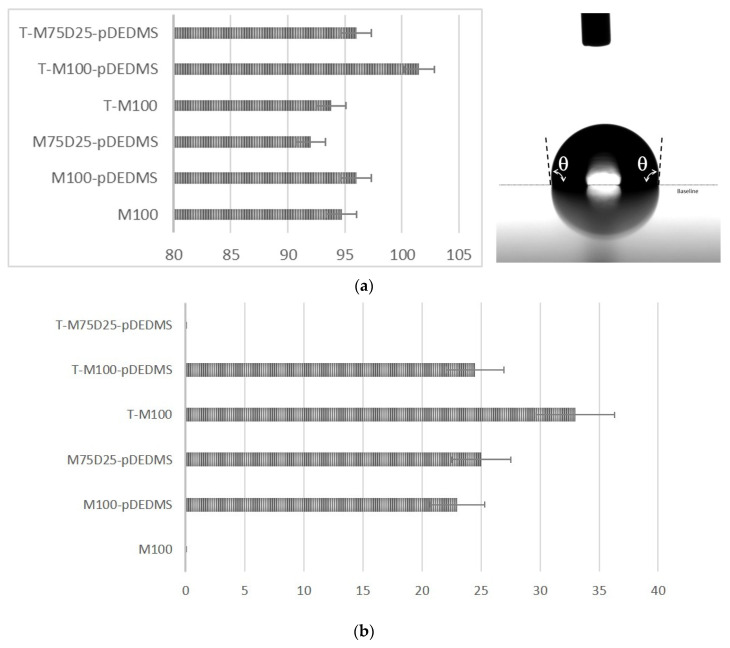
(**a**) Static contact angle θ (°) for the six coatings and typical image measured for M100-pDEDMS coating. (**b**) Dynamic contact angle hysteresis Δθ (°) for the six coatings.

**Figure 6:**
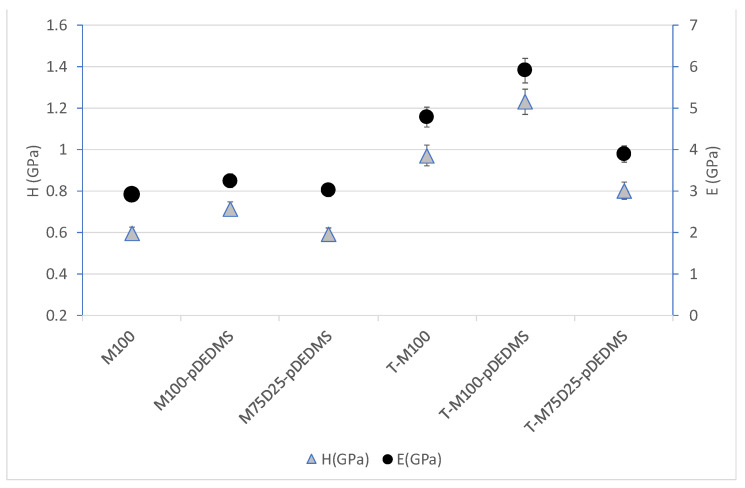
Hardness H and elastic modulus E for all coatings.

The hydrophobic and crack resistance properties were now investigated, and the results are reported in Figure 5 and Figure 6.

### 3.4. Hydrophobicity of DEDMS/MTMS Coatings

In this study, the incorporation of DEDMS was intended to improve the hydrophobic properties of the coatings. The presence of type D^2^ silicon atoms (SiC_2_O_2_) reduces the number of residual Si-OH bonds in the siloxane network, which is observed in NMR with the evolution of the T^2^:T^3^ ratio in the presence of DEDMS or p-DEDMS, increasing the hydrophobic character. The contact angles in static mode are not very different in the series of six samples and remain around 95° except for the T-M100-pDEDMD sample for which it reaches 102°, characterizing hydrophobic coatings. Among these values, it is worth noting the contact angle value of the coating containing only MTMS. In fact, this very high value for this coating composed of 100% MTMS is among the highest contact angle values described for this type of coating. It can be compared to the value of 76° described by Juan-Diaz et al. [29]. This very hydrophobic character is explained in our case by the very high condensation rate measured by ^29^Si NMR. Moreover, this hydrophobic character is preserved for all the studied coatings.

In addition to static contact angle measurements, dynamic contact angle measurements were performed to compare the hydrophobicity of the sol-gel coatings T-100, T-M100-pDEDMS and T-M75-D25-pDEDMS to previous measurements on free-TEOS coatings. Figure 5a,b present, respectively, the results obtained in statics and dynamics. Hysteresis was not measurable for the M100 formulation and for T-M75-D25 –pDEDMS. For the first coating, this is probably due to the presence of cracks leading to water penetration into the coating, while for the second, a higher sample roughness can be considered. The addition of silicone oil in the form of p-DEDMS has a great impact on the hydrophobic behavior, since a low contact angle hysteresis was measured for both formulations M100-pDEDMS and M75-D25-pDEDMS, the first being the lowest with a Δθ value less than 25° as compared to the M-100 reference formulation. A similar hysteresis angle between the two formulations M100-pDEDMS and M75-D25-pDEDMS indicates that the introduction of DEDMS as a precursor does not change the coating properties as drastically as the p-DEDMS does (both for hydrophobicity and cracking).

### 3.5. Mechanical Properties of DEDMS/MTMS Coatings

To evaluate the mechanical properties of these coatings, a quantitative analysis was carried out through nanoindentation tests. The hardness and elastic modulus of all the formulations are presented in Figure 6.

When TEOS precursor is added (Figure 6), it appears that the hardness and elastic modulus increase (40% increasing) for all coatings containing TEOS compared to formulations without TEOS. This effect can be linked to the increase in inorganic network fraction and to the formation of quaternary silicon-oxygen tetrahedra “Q” in the silicate structure, as seen in Table 3. This could lead to a stiffer network, and it is in good agreement with the literature. Bautista et al. [17] have indeed shown that a 20% addition of TEOS to a methacryloxypropyltrimethoxysilane (MAPTMS)-based sol-gel coating leads to an increase in both hardness (0.60 to 0.81 GPa) and elastic modulus (4.5 to 6.3 GPa).

The DEDMS precursor in substitution of the MTMS led to a decrease in the mechanical properties (both E and H) of the coating. This tendency is particularly noticeable in the TEOS formulation with a decrease of about 20%. This effect could be due to the fact that the partial substitution of the MTMS with DEDMS leads to either a lower degree of condensation (less stiff network) or a higher organic content due to the additional methyl group present in the DEDMS precursor and thus in the obtained film. As seen in Table 4, regarding whether the degree of condensation is similar or there is a light increase for M75-D25-pDEDMS and T-M75-D25-pDEDMS compared to M100-pDEDMS and M100-pDEDMS, data tend to support the latter hypothesis.

More surprisingly, precondensed DEDMS addition seems to have a slight increasing effect on the hardness and the elastic modulus of the obtained coatings. The introduction of precondensed DEDMS, as in M100-pDEDMS, increases the Young’s modulus from 3 to 4 GPa and the hardness from 0.55 to 0.70 GPa as compared to the reference M100 coating. In that respect, the T-M100-pDEDMS presents the higher mechanical properties of all coatings.

## 4. Conclusions

**Figure 7:**
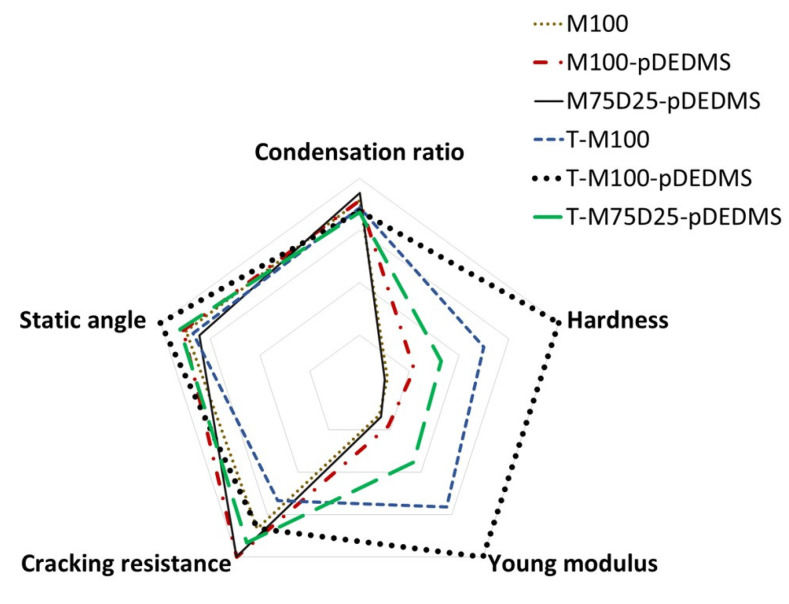
Correlation between the formulation and the functional properties.

Sol-gel coatings with a silicon oil addition have been carried out in the mixed MTMS-DEDMS-TEOS system. The aim was to modulate jointly or separately the mechanical properties and hydrophobicity of the coatings. First of all, the beneficial effect of silicon oil has been highlighted. In fact, there is a significative difference between in situ generated or precondensed DEDMS. In the first case, the effect is neutral but in the second one, there is a major effect both on the limitation of cracking and also on the hydrophobic properties. The formulation with TEOS, MTMS and only 2 wt.% of precondensed DEDMS silicon oil has shown the best hydrophobic behavior. The presence of TEOS in the coating also improves the mechanical properties by increasing the hardness and the Young modulus following the same evolution. To improve the comprehension of the mechanisms at a microscopic scale, the integration of silicon oil has been followed by NMR analysis. The addition of DEDMS in the sol-gel network has a negative effect on the microstructure and does not improve the mechanical and hydrophobic properties. Relative to TEOS, the increase in the mechanical properties is proved. The presence of this precursor in the system acts on the decrease in MTMS condensation degree, but the beneficial effect of TEOS condensation results in a stiffening of the global system. A similar conclusion can be drawn on the effect of pDEDMS. Even in a very small proportion (2 wt.%), T-M100-pDEDMS compared to T-M100 presents a higher condensation degree of TEOS (91% vs. 85%, respectively), which explains that with pDEDMS, the stiffening increases and the mechanical properties H and E also increase. To evidence the correlation existing between the formulation (based on the three precursors) and the properties, a radar diagram was drawn, as shown in Figure 7. It reports the behavior of the 6 sol-gel systems with respect to the following properties: condensation ratio, hardness, Young’s modulus, hydrophobicity (static contact angle) and resistance to cracking. In summary, these three precursors have synergistic effects and make it possible to modulate the functionalities of coatings.

## Figures and Tables

**Table 1:** Role and semi-developed formula of silane precursors.

Precursors	Role	Structure
TEOS	Silica generation and siloxane network	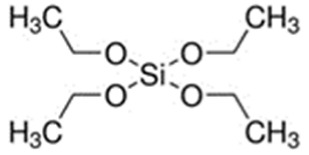
MTMS	Hybrid siloxane network precursor	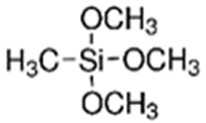
DEDMS	Hybrid siloxane network precursor and/or in situ oil generation	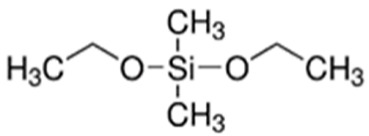

**Table 2:** Nomenclature and molar compositions of the different sols ^1^ and coatings.

	MTMS	H_2_O	CH_3_CO_2_H	TEOS	DEDMS	p-DEDMS		
						DEDMS	H_2_O	CH_3_CO_2_H
M100	1	2.6	0.1					
M100-pDEDMS	1	2.6				0.034	0.17	0.17
M75-D25-pDEDMS	1	4.0			0.3	0.051	0.26	0.26
T-M100	1	9.7	0.2	0.9				
T-M100-pDEDMS	1	9.7		0.9		0.09	0.42	0.42
T-M75-D25-pDEDMS	1	12.9		1.1	0.3	0.12	0.61	0.61

^1^ The solvent is systematically composed of 2:1 ratio of 2-propanol and 2-butoxyethanol, respectively.

**Table 3:** Data extracted from deconvolution of ^29^Si MAS NMR spectra of the different hybrid materials.

	δ in ppm (Ratio in %)								
	D^1^	D^2^	D^2^	T^2^	T^3^	Q^2^	Q^3^	Q^3^	Q^4^
M100				−58.2	−67.1				
				(19)	(81)				
M100-pDEDMS		−19.4	−21.8	−57.9	−67.0				
		(3)	(2)	(18.5)	(76.4)				
M75-D25-pDEDMS		−21.4	−22.8	−58.3	−67.3				
		(19.6)	(1.3)	(10.2)	(68.9)				
T-M100				−56.3	−65.1		−98.6	−102.3	−110.1
				(13.2)	(39)		(7.8)	(12.2)	(27.7)
T-M100-pDEDMS	−11.3	−18.5		−57.2	−65.6	−94.1		−101.9	−110.3
	(1.3)	(5.7)		(14.5)	(37.1)	(1.1)		(12.2)	(28.1)
T-M75-D25-pDEDMS	−13.0	−19.1	−22.9	−57.0	−65.4	−92.9		−101.4	−110.2
	(3)	(8.5)	(0.9)	(12.2)	(29.4)	(1.7)		(13.6)	(30.7)

**Table 4:** Degree of condensation of siloxane networks.

	Degree of Condensation (% ± 0.5%)		
	DEDMS	MTMS	TEOS
	(D^1^ + 2D^2^)/2(D^1^ + D^2^)	(2T^2^ + 3T^3^)/3(T^2^ + T^3^)	(2Q^2^ + 3Q^3^ + 4Q^4^)/4(Q^2^ + Q^3^ + Q^4^)
M100		93.7	
M100-pDEDMS	100	93.5	
M75-D25-pDEDMS	100	95.7	
T-M100		91.6	85.4
T-M100-pDEDMS	91	90.6	91.3
T-M75-D25-pDEDMS	100	90.2	90.8

## Data Availability

Data are contained within the article and supplementary materials.

## Supplementary Materials

The following supporting information can be downloaded at https://www.mdpi.com/article/10.3390/ma17020368/s1, Figure S1: ^29^Si{^1^H} NMR spectra of precondensed DEDMS after 4 h in acetic acid medium. Figure S2: Young’s modulus E and hardness H as a function of penetration depth, measured by sinus mode indentation (maximum normal force of 10 mN, loading and unloading rate of 20 mN·min^−1^, sinus amplitude of 0.1 mN, sinus frequency of 12 Hz). Figure S3: Normal load as a function of penetration depth during static nanoindentation test (maximum normal load of 2 mN, loading and unloading rate of 4 mN·min^−1^) measured for T-M100-pDEDMS sample.
