# Supplementary material for: Development of Multifunctional Hybrid Coatings (Mechanically Resistant and Hydrophobic) Using Methyltrimethoxysilane–Diethoxydimethylsilane–Tetraethoxysilane Mixed Systems
Source: Materials (Basel). 2024 Jan 11;17(2):368. doi: 10.3390/ma17020368 (PMC10817560; doi:10.3390/ma17020368)
Supplement: Supplementary file 1 [file materials-17-00368-s001.zip › materials-2797040-supplementary.pdf]

materials

Development of Multifunctional Hybrid Coatings (Mechanically Resistant and Hydrophobic) Using Methyltrimethoxysilane–Diethoxydimethylsilane–Tetraethoxysilane Mixed Systems

Charlène Pellegrini, Sandrine Duluard, Marie Gressier, Viviane Turq, Florence Ansart and Marie-Joëlle Menu *

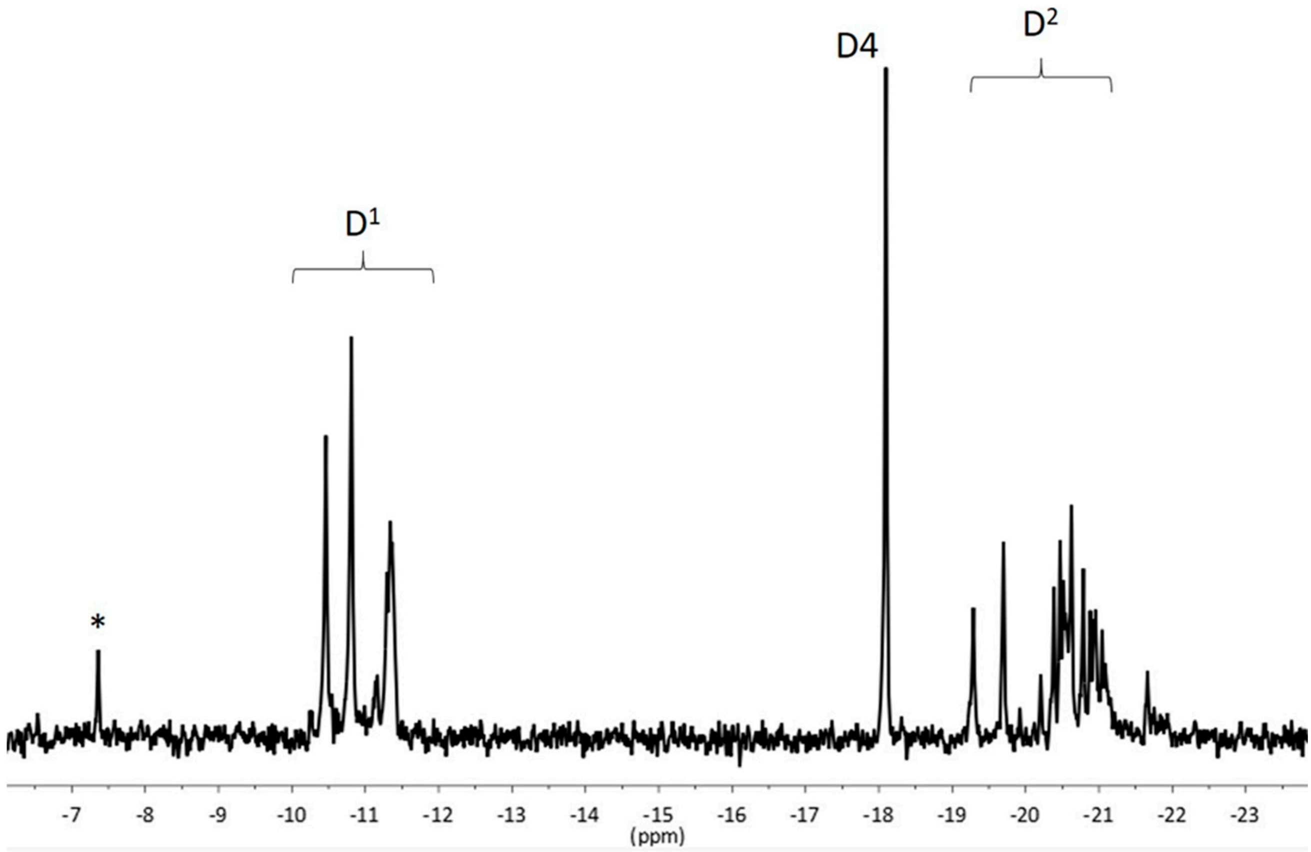

Figure S1. $^{29}Si\{^1H\}$ NMR spectra of precondensed DEDMS after 4 hours in acetic acid medium.

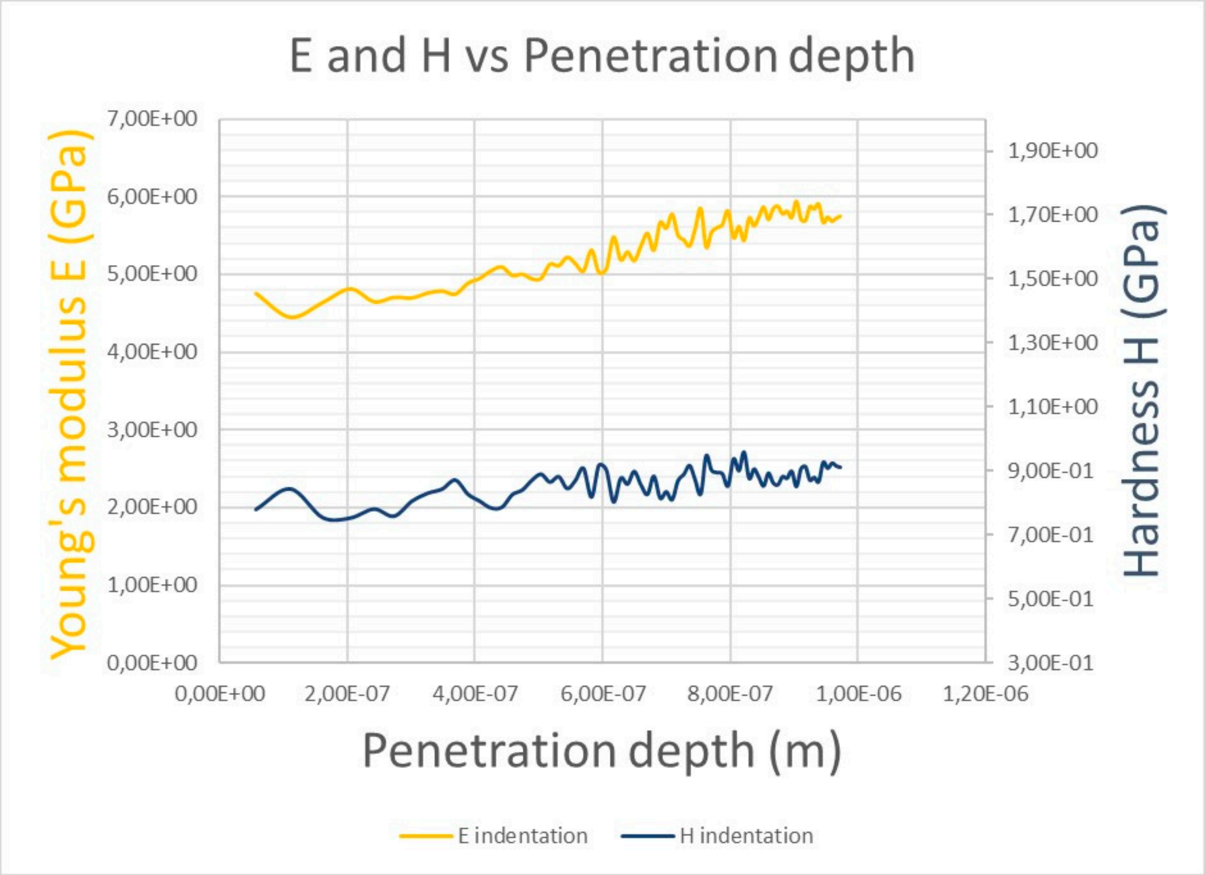

Figure S2. Young's modulus E and hardness H as a function of penetration depth, measured by sinus mode indentation (maximum normal force of 10 mN, loading and unloading rate of 20 mN·min^{-1}, sinus amplitude of 0.1 mN, sinus frequency of 12 Hz).

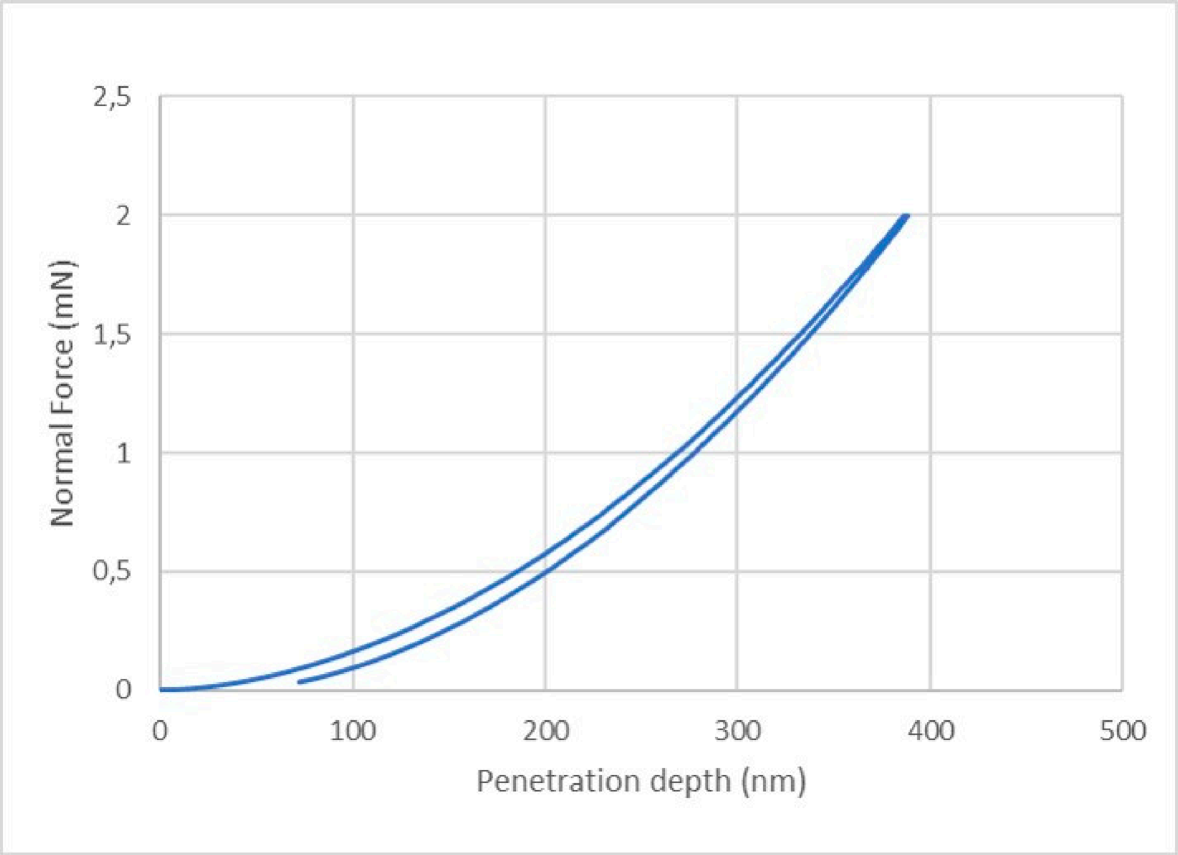

Figure S3. Normal load as a function of penetration depth during static nanoindentation test (maximum normal load of 2 mN, loading and unloading rate of 4 mN·min^{-1}) measured for T-M100-pDEDMS sample.
